# Wild poliovirus type 3 (WPV3)-shedding event following detection in environmental surveillance of poliovirus essential facilities, the Netherlands, November 2022 to January 2023

**DOI:** 10.2807/1560-7917.ES.2023.28.5.2300049

**Published:** 2023-02-02

**Authors:** Erwin Duizer, Wilhelmina LM Ruijs, AD Putri Hintaran, Mariska C Hafkamp, Margreet van der Veer, Margreet JM te Wierik

**Affiliations:** 1 Centre for Infectious Diseases Control, National Institute for Public Health and the Environment (RIVM), Bilthoven, the Netherlands; 2 Public Health Service Region Utrecht, Zeist, the Netherlands; 3 National Authority for Containment, Health and Youth Care Inspectorate (IGJ), Utrecht, the Netherlands

**Keywords:** poliovirus, wild poliovirus type 3 (WPV3), Saukett strain G, environmental surveillance, containment, Global Action Plan (GAP), poliovirus essential facility (PEF)

## Abstract

On 21 November 2022, a wild poliovirus type 3 (WPV3) was isolated from an environmental surveillance sample of poliovirus essential facilities in the Netherlands. All 51 employees with access to this strain were screened for ongoing or recent poliovirus infection. One employee shedding WPV3 was identified on 8 December and placed in isolation; monitoring and contact tracing were initiated. WPV3 shedding continued for 4 weeks and stopped 5 January 2023. Isolation was lifted 11 January and no further transmission was detected.

The near complete eradication of wildtype polioviruses (WPV) means that strict containment by facilities for essential work with infectious WPV is required. In the Netherlands, we have implemented environmental surveillance around all poliovirus essential facilities (PEFs) premises to monitor possible breaches of containment. After the isolation and identification of WPV3 Saukett G strain in a sewage sample collected on 15 November 2022 by the National Polio Laboratory (NPL) in the Netherlands, an immediate response was required to assess any possible ongoing WPV3 shedding and mitigate the risk. Here we describe this response, including the isolation of WPV3 in a sewage sample, and identification, isolation and monitoring, as well as tracing of contacts of an infected employee.

## Environmental surveillance

Routine environmental surveillance at the PEFs is ordered by the national authority for containment of poliovirus (NAC) and performed by the NPL in the Netherlands since 2020. Sampling sites collect wastewater from buildings used for vaccine production, polio diagnostic laboratories and toilet groups. Analysis of PEF samples is based on virus isolation to detect infectious polioviruses; any infectious poliovirus isolation from wastewater indicates a breach of containment in a PEF. The release and detection of inactivated viruses and incomplete genomic fragments in wastewater by PCR does not necessarily indicate the presence of infectious virus, nor does it constitute a risk, and therefore is not regulated.

**Figure 1:**
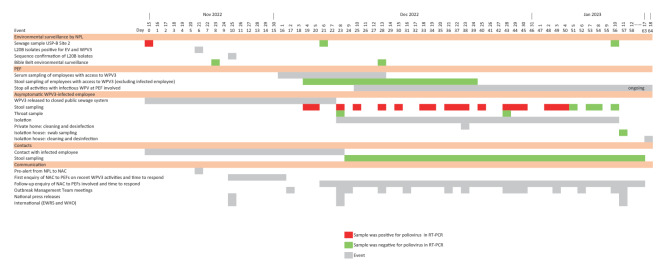
Timeline of surveillance and response to a wild poliovirus type 3 (WPV3)-shedding event at a poliovirus essential facility, Bilthoven, Netherlands 15 November 2022–18 January 2023

EV: enterovirus; EWRS: Early Warning and Response System; NAC: national authority for containment of poliovirus; NPL: national polio laboratory; PEF: poliovirus essential facility; USP: Utrecht Science Park; WHO: World Health Organization; WPV: wild poliovirus.

During 2022, sewage samples were collected and analysed every 3 weeks. A total of 74 PEF samples were analysed, of which 50 were from the three sampling sites at the Utrecht Science Park-Bilthoven (USP-B); 18 of these were from the implicated USP-B Sample Site 2. All samples were negative for infectious poliovirus except one. The positive sample, collected on 15 November 2022 at USP-B Site 2, yielded two isolates from L20B cell culture (Figure 1). Whole genome sequencing was performed on these two isolates, which yielded the WPV3-Saukett G strain with two and three mutations compared with the vaccine seed stocks used by the PEF. Two follow-up samples from USP-B Site 2, collected on 6 December 2022 and 10 January 2023, tested negative for infectious poliovirus.

L20B (mouse cell line expressing the gene for the human cellular receptor for poliovirus) cell cultures were used for poliovirus isolation according to the World Health Organization protocol [6]. Surveillance performed in the Bible belt [3] included the residence of the infected employee.

## Response to a poliovirus isolation from a PEF sewage system

**Figure 2:**
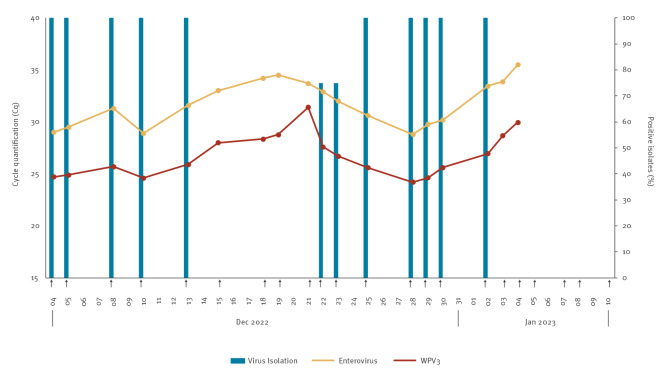
Enterovirus and poliovirus detection of stool samples of a wild poliovirus 3-infected employee, Bilthoven, Netherlands, 4 December 2022–10 January 2023 (n = 22 samples)

As per national and international guidelines [1,2], the NAC and PEF investigated possible incidents/accidents (Figure 1) among employees and the PEF initiated a root cause analysis to identify the process in which the virus had escaped containment. Identification of possible ongoing shedding was a main priority; since the isolated WPV3 strains already had 2 and 3 mutations, this would suggest human shedding instead of direct release from the PEF. Two stool samples and one serum sample were requested from each employee with access to WPV3 strains (n = 51) in the 3 weeks preceding the positive sample isolation. Stools (n = 96, at least one sample collected per person) were analysed using in-house RT-PCR assays for the detection of enterovirus (EV) and WPV3 (Saukett-specific test). All employees were vaccinated and in one employee, a serological response to a recent infection was found. The two stool samples from this employee tested positive for EV and WPV3 (Figure 1 and 2). All stool samples from the other 50 employees tested negative.

WPV3: wild poliovirus 3.

Enterovirus and WPV3 detection by RT-PCR and virus isolation (per cent positive of four cultures). All sampling dates (black arrows) are indicated on the X axis. If no data are shown, the detection was negative.

## Public health response

Starting 8 December 2022, the infected employee agreed to voluntary isolation under daily supervision of the local public health service. Because this employee resided in an area of the Netherlands with suboptimal (< 90%) vaccination coverage [3], home isolation was not an option. For the duration of the isolation, the employee was housed in a residence provided by the PEF, which was specially designed for isolation of employees and located in an area with > 90% vaccination coverage. The infected employee was instructed by the local public health service to follow strict hygiene measures, and all stools were collected in a disposable system as per containment guidelines in the Netherlands [1,3]. Waste requiring containment according to World Health Organization (WHO) Global Action Plan IV (GAPIV) [4] (i.e. all waste suspected to contain infectious WPV3) was packed, transported and incinerated according to national guidelines [1]. 

The isolation of the employee and monitoring of virus shedding continued throughout the holiday season including Christmas and New Year’s Day (Figure 1). Receiving guests in the isolation residence was not allowed, but the employee could walk, exercise or meet people outside without physical contact. Since being away from home and living with restrictions for a long period can be a psychological burden, the psychological and mental health of this employee was also closely monitored and psychological support was provided by the public health service and PEF during the isolation period. After 33 days, the employee exited isolation on 11 January 2023, after three consecutive negative stool samples. 

## Diagnostic results of the infected employee

When the infected employee entered isolation on 8 December, and when the virus load in stool increased on 13 December, a throat swab was collected. The swabs tested negative for EV and WPV3, indicating that oral virus excretion via breathing and talking was unlikely, and only stringent hand and toilet hygiene measures would be required to prevent secondary transmission. Monitoring of stool samples during the isolation period showed the presence of EV/WPV3 between 4 December 2022 and 4 January 2023, as shown in Figure 2.

The time between virus presence in sewage (15 November 2022) and end of virus excretion from the employee (5 January 2023) was 51 days. We do not yet have an explanation for the rather long shedding by the employee; the serological response to PV3 was high and IgA deficiency was excluded.

Full genome sequences were obtained from the two sewage isolates and two stool samples of the infected employee from 5 and 8 December. The genomes of the sewage isolates and the stool samples were not identical but had one common mutation (C3082T, numbering as per GenBank ref: KP247597) relative to the vaccine strain, indicating that the identified shedding employee was indeed the most likely source of the virus isolated from the sewage.

## Contact tracing

Following identification of a WPV3-shedding employee, the PEF and public health services started identifying contacts. In particular, PEF colleagues who worked in close contact and/or those who shared the same toilet area at the workplace were included (n = 16). Eleven private contacts were identified (visitors/visitee for more than 4 h). A total of 54 stool samples from the 27 contacts tested negative for EV and WPV3.

## Discussion

We report an asymptomatic WPV3 infection in a fully vaccinated PEF employee, which was discovered following WPV3 detection in the routine environmental surveillance of PEFs in the Netherlands. The isolation of WPV from a sewage sample indicated an unnoticed breach of containment at the facility and triggered an immediate response, quickly identifying the WPV-shedding employee and prompting rapid public health action. 

The World Health Organization published the third edition of the WHO Global Action Plan (GAPIII [5]) on Poliovirus Containment in 2015, and the fourth edition in 2022 (GAPIV [4]). To continue working with infectious polioviruses, PEFs must be GAP-certified by the NAC. A PEF must show that their activities are deemed essential and that they comply to the safeguards described in GAPIII or IV. PEFs can be facilities for vaccine production or polio diagnostics and surveillance. The primary safeguard requires strict poliovirus containment by the PEF, including proof of vaccination from all employees, the secondary safeguard requires > 90% polio vaccination coverage around the PEF, and the tertiary safeguard requires environmental controls in place to minimise onward transmission risk after release of PV from a PEF [4,5]. The first and most important safeguard is containment of infectious polioviruses by the PEF. In the Netherlands, we implemented environmental surveillance around all PEF premises in 2020 and have since detected three WPVs.

We found no signs for ongoing transmission, and the swab samples taken from the infected employee’s isolation residence were all negative for WPV3. This shows that stringent toilet and hand hygiene were sufficient to mitigate the risk in this case, where no shedding via the throat (oral) was detected.

Even though intermittent shedding of poliovirus has been reported for poliovirus infections [6,7], information for WPV3 is limited, and intermittent shedding is more likely continued shedding at a low concentration that is below the detection limit of the method used. With the development of more sensitive methods, detection of intermittent shedding becomes less likely. Here we show intermittent shedding, based on the WHO algorithm for acute flaccid paralysis (AFP) cases (i.e. virus isolation on two L20B and two RD tubes [6,8]) and, using molecular methods, show that shedding did actually continue. Our findings show that the current algorithm for detecting or excluding poliovirus infection in AFP cases is not the most sensitive method to determine the end of poliovirus shedding in vaccinated and infected persons.

## Conclusions

This event shows that incidents that lead to a breach of containment and even an infection can remain unnoticed and not reported if routine monitoring is not in place. This case clearly shows that environmental surveillance is an essential tool to detect unnoticed breaches of containment and personnel infection at PEFs. We believe our environmental surveillance strategy has proven very valuable and strongly propose that other countries implement a similar system.
